# Inhibition of transient receptor potential cation channel 6 promotes capillary arterialization during post‐ischaemic blood flow recovery

**DOI:** 10.1111/bph.15942

**Published:** 2022-10-03

**Authors:** Takuro Numaga‐Tomita, Tsukasa Shimauchi, Yuri Kato, Kazuhiro Nishiyama, Akiyuki Nishimura, Kosuke Sakata, Hiroyuki Inada, Satomi Kita, Takahiro Iwamoto, Junichi Nabekura, Lutz Birnbaumer, Yasuo Mori, Motohiro Nishida

**Affiliations:** ^1^ National Institute for Physiological Sciences (NIPS) National Institutes of Natural Sciences Aichi Japan; ^2^ Exploratory Research Center on Life and Living Systems (ExCELLS) National Institutes of Natural Sciences Aichi Japan; ^3^ SOKENDAI (School of Life Science The Graduate University for Advanced Studies) Aichi Japan; ^4^ Shinshu University School of Medicine Nagano Japan; ^5^ Graduate School of Pharmaceutical Sciences Kyushu University Fukuoka Japan; ^6^ Graduate School of Medical Sciences Kyushu University Fukuoka Japan; ^7^ Faculty of Medicine Fukuoka University Fukuoka Japan; ^8^ Faculty of Pharmaceutical Sciences Tokushima Bunri University Tokushima Japan; ^9^ NIEHS, NIH Research Triangle Park North Carolina USA; ^10^ Institute for Biomedical Research (BIOMED) Catholic University of Argentina Buenos Aires Argentina; ^11^ Graduate School of Engineering Kyoto University Kyoto Japan

**Keywords:** channel, peripheral arterial disease, phosphorylation, transient receptor potential, vascular smooth muscle cell

## Abstract

**Background and Purpose:**

Capillary arterialization, characterized by the coverage of pre‐existing or nascent capillary vessels with vascular smooth muscle cells (VSMCs), is critical for the development of collateral arterioles to improve post‐ischaemic blood flow. We previously demonstrated that the inhibition of transient receptor potential 6 subfamily C, member 6 (TRPC6) channels facilitate contractile differentiation of VSMCs under ischaemic stress. We here investigated whether TRPC6 inhibition promotes post‐ischaemic blood flow recovery through capillary arterialization *in vivo*.

**Experimental Approach:**

Mice were subjected to hindlimb ischaemia by ligating left femoral artery. The recovery rate of peripheral blood flow was calculated by the ratio of ischaemic left leg to non‐ischaemic right one. The number and diameter of blood vessels were analysed by immunohistochemistry. Expression and phosphorylation levels of TRPC6 proteins were determined by western blotting and immunohistochemistry.

**Key Results:**

Although the post‐ischaemic blood flow recovery is reportedly dependent on endothelium‐dependent relaxing factors, systemic TRPC6 deletion significantly promoted blood flow recovery under the condition that nitric oxide or prostacyclin production were inhibited, accompanying capillary arterialization. Cilostazol, a clinically approved drug for peripheral arterial disease, facilitates blood flow recovery by inactivating TRPC6 via phosphorylation at Thr69 in VSMCs. Furthermore, inhibition of TRPC6 channel activity by pyrazole‐2 (Pyr2; BTP2; YM‐58483) promoted post‐ischaemic blood flow recovery in *A* *polipoprotein* E‐knockout mice.

**Conclusion and Implications:**

Suppression of TRPC6 channel activity in VSMCs could be a new strategy for the improvement of post‐ischaemic peripheral blood circulation.

## Graphical abstract

*Transient receptor potential canonical 6* (*T* *rpc* *6*)‐knockout (KO) (C6(−/−)) mice has better blood flow recovery after hindlimb ischaemia than wild type (C6(+/+)) mice. Vascular smooth muscle‐specific rescue of TRPC6 expression reverses that. *P < 0.05. TRPC6 inhibition by Pyr2 facilitates blood flow recovery after hindlimb ischaemia in hypercholesterolemic *Apo*E‐KO mice. *P < 0.05.

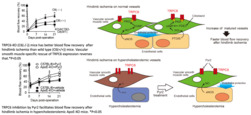

**What is already known**

- TRPC6 negatively regulates vascular smooth muscle cells (VSMCs) differentiation *in vitro*.

**What does this study add**

- Endothelium‐dependent phosphorylation of TRPC6 in VSMCs contributes to endogenous stress resistance against hindlimb ischaemia.

**What is the clinical significance**

- Suppression of TRPC6 channel activity promotes post‐ischaemic blood flow recovery through capillary arterialization.

**Abbreviations**
*ACTA2*
: actin alpha 2, smooth muscle, aorta
α‐SMA: α‐smooth muscle actin
*Apo*E: *Apolipoprotein* E
EDH: endothelium‐dependent hyperpolarization
EDHF: endothelium‐derived hyperpolarizing factor
PDGF: platelet derived growth factor
Pyr2: pyrazole‐2; BTP2; YM584893
TGF‐β: transforming growth factor β
VSMCs: vascular smooth muscle cells

## 1 INTRODUCTION

Ischaemic diseases induced by progressive occlusion of large conductance artery after atherosclerosis are the leading causes of morbidity and death in the developed countries. Our body has intrinsic compensatory mechanisms against arteriosclerotic occlusion of a major artery (Schaper, 2009), including growth and development of new sprouting vessels from pre‐existing capillaries or major arteries in the ischaemic tissue (angiogenesis) (Haas et al., 2012), recruitment of synthetic vascular smooth muscle cells (VSMCs) and the coverage of pre‐existing or nascent capillary vessels with VSMCs (capillary arterialization) and growth and development of collateral arterioles (arteriogenesis) (Faber et al., 2014). A great deal of effort has been poured into attenuate the consequences of tissue ischaemia by promoting angiogenesis using growth factors, such as vascular endothelial growth factors and fibroblast growth factors, or inducing inflammatory response using monocyte chemotactic protein 1, while receptors for these growth factors and chemokines have been down‐regulated within 7 days after ischaemia (Fung & Helisch, 2012; Haas et al., 2012). In addition, blood perfusion of post‐ischaemic peripheral tissues, including gastrocnemius, is not correlated well with the increase of capillary densities (Couffinhal et al., 1998; Sullivan et al., 2002). Therefore, post‐angiogenic processes of vessel maturation are likely to be more critical for the formation of functional microcirculation than angiogenesis (LeBlanc et al., 2012).

Central part of vessel maturation is recruitment and coverage of nascent or pre‐existing capillaries by perivascular cells, in which VSMCs play a critical role (LeBlanc et al., 2012). VSMCs can switch back and forth between highly proliferative synthetic and fully differentiated contractile phenotypes, and contribute to reconstitution of vasculature after tissue injury (Bennett et al., 2016; Davis‐Dusenbery et al., 2011; Frismantiene et al., 2018). Abnormal phenotypic switching of VSMCs is a hallmark of vascular disorders such as atherosclerosis and restenosis after angioplasty (Bennett et al., 2016; Davis‐Dusenbery et al., 2011; Frismantiene et al., 2018). The recruitment and subsequent differentiation of VSMCs are regulated by endothelial cells. Endothelial cells secrete platelet derived growth factor (PDGF) to promote VSMC proliferation/migration and transforming growth factor β (TGF‐β) to promote VSMC differentiation, respectively (LeBlanc et al., 2012). Furthermore, vasodilatory mediators released from endothelial cells are involved in collateral development after hindlimb ischaemia, such as nitric oxide (NO), prostacyclin (PGI_2_) and endothelium‐derived hyperpolarizing factor (EDHF) (Otsuka et al., 2006; Park et al., 2010; Troidl et al., 2010; Waldron & Cole, 1999). However, how endothelial cell‐derived factors promote capillary arterialization is not comprehensively understood because common target molecules for endothelial cell‐derived factors have not been identified.

Cilostazol is a reversible phosphodiesterase III inhibitor that is clinically used for peripheral arterial disease (PAD) patients with intermittent claudication (Kherallah et al., 2021). Cilostazol shows pleiotropic effects, such as antiplatelet and vasodilatory actions. Cilostazol has been reported to promote angiogenesis by increasing the release of endothelial growth factors and the activities of endothelial NO synthase (eNOS) and peroxisome proliferator‐activated receptor γ (PPAR‐γ; NR1C3; Biscetti, Pecorini, Arena, et al., 2013; Biscetti, Pecorini, Straface, et al., 2013; Hori et al., 2012), but how cilostazol potently promotes post‐ischaemic blood flow recovery remains unknown. We previously reported that the vasodilatory effect of cilostazol is mediated by protein kinase A (PKA)‐mediated phosphorylation of TRPC6 at 69th threonine residue (Thr69). The phosphorylation at Thr69 negatively regulates TRPC6 channel activity, thereby inhibiting angiotensin II‐stimulated Ca^2+^‐dependent response in rat aortic VSMCs (Nishioka et al., 2011).

Transient receptor potential canonical (TRPC) subfamily proteins are the molecular entity of receptor‐activated Ca^2+^‐permeable channels in vertebrates. Especially, diacylglycerol‐sensitive TRPC members, TRPC3 and TRPC6, reportedly contribute to receptor‐stimulated cardiovascular remodelling (Nishida et al., 2020; Onohara et al., 2006). In the vasculature, TRPC6 is highly expressed in VSMCs and reportedly involved in pathological remodelling of pulmonary VSMCs caused by hypoxic stress (Smith et al., 2015). We have reported that the genetic ablation of *trpc6* facilitates VSMC differentiation without affecting cell proliferation and migration properties in primary‐cultured mouse aortic VSMCs. Furthermore, TRPC6 contributes to ischaemic stress‐induced suppression of VSMC differentiation (Numaga‐Tomita et al., 2019). These data prompted us to investigate whether TRPC6 participates in the process of vessel maturation after hindlimb ischaemia in mice.

In this study, we examined the role of TRPC6 channel activity in the post‐ischaemic blood flow recovery of hindlimb ischaemia mice. We demonstrate that systemic TRPC6 deletion facilitated blood flow recovery after hindlimb ischaemia under the condition that nitric oxide or prostacyclin production was inhibited. By using transgenic mice, we revealed that cilostazol facilitates blood flow recovery by suppressing TRPC6 via phosphorylation at Thr69 in VSMCs. Inhibition of TRPC6 channel activity by pyrazol‐2 (Pyr2; BTP2; YM‐58483) improved blood flow recovery after hindlimb ischaemia in *Apolipoprotein* E‐knockout (*Apo*E‐KO) mice, suggesting a new therapeutic potential of TRPC6 inhibition for the treatment of peripheral arterial disease patients with hypercholesterolemia.

## 2 METHODS

### 2.1 Compliance with requirements for studies using animals

#### 2.1.1 Validity

129Sv and C57BL/6 background mice were used in this study. *Trpc6* knockout (TRPC6(−/−)) and its littermate control (TRPC6(+/+))129Sv mice were provided by Dr. Birnbaumer in NIEHS. The level of blood flow recovery after hindlimb ischaemia reportedly depends on the mice strain (Helisch et al., 2006). 129Sv has medium recovery rate compared to C57BL/6 or Balb/C mice. As an endothelial dysfunction model, we utilized C57BL/6 background *Apo*E‐KO mice.

#### 2.1.2 Ethical statement

All protocols using 129Sv background TRPC6(+/+), TRPC6(−/−), KO/*Acta2*‐C6(WT) and KO/*Acta2*‐C6(T69A) mice and C57BL/6 background *Apo*E‐KO mice were reviewed and approved by the ethic committees at National Institutes of Natural Sciences or the Animal Care and Use Committee, Kyushu University, and were performed according to the institutional guidelines concerning the care and handling of experimental animals (protocol code: 21A057 approved on 29 March 2021, 22A026 approved on 31 March 2022, A19‐279‐1 approved on 7 October 2020, and A21‐071‐0 approved on 19 February 2021). Animal studies are reported in compliance with the ARRIVE guidelines (Percie du Sert et al., 2020) and with the recommendations made by the *British Journal of Pharmacology* (Lilley et al., 2020).

#### 2.1.3 Animals

Eight‐ to 10‐week‐old male mice (20‐ to 22‐g body weight) were used in this study. All 129Sv mice were maintained and bred in the animal facilities in the National Institute of Natural Sciences and Kyushu University. C57BL/6 control and *Apo*E‐KO mice were purchased from SLC (Shizuoka, Japan). We constructed two transgenes by inserting wild‐type TRPC6 (WT) or mutant TRPC6 (T69A) cDNA between the human actin, alpha 2, smooth muscle, aorta (*Acta2*) promoter and the SV40 polyadenylation sequence of the plasmid (Miwa et al., 2000). Each transgene was microinjected into the pronuclei of fertilized TRPC6(‐/‐) mouse embryos at the single‐cell stage. We implanted the embryos into pseudopregnant foster mothers. Genotyping for TRPC6(‐/‐) mice has been described previously (Dietrich et al., 2005). Genotyping for *Acta2*/TRPC6‐harbouring mice was performed using PCR primers; *Acta2* promoter‐forward 5′‐TGCTCCAGCCTTTCTAATTTTATG‐3′ and TRPC6‐reverse 5′‐CTGGTCCTCGATTAGCTAACCTTCT‐3′. The PCR was performed by Phusion High‐Fidelity DNA polymerase (Thermo Scientific).

#### 2.1.4 Housing and husbandry

Mice were maintained in specific‐pathogen‐free area and housed individually or in groups of no more than five animals per cage under controlled environmental conditions (12‐h light–dark cycle, room temperature of 22–23°C and humidity 50%–60%) and given free access to food and water. Humane endpoint was set as follows:‐ (1) more than 20% weight loss compared to the weight of age‐matched mice, (2) crouching for more than 48 h and (3) inability of food and water intake. In addition, walking ability and tissue necrosis as signs of poor prognosis were carefully monitored. Walking ability was almost recovered in a few days after the surgery and there were no signs of necrosis and weight loss due to the defect of food and water intake.

#### 2.1.5 Experimental procedures

Mice were anaesthetized with intraperitoneal injection of medetomidine (0.3 mg·kg^−1^), midazolam (4 mg·kg^−1^) and butorphanol (5 mg·kg^‐1^) with spontaneous breathing and body temperature was maintained using heat pad (38°C). Under this condition, unilateral hindlimb ischaemia was induced by ligation of left femoral arteries to 129Sv mice as described previously (Helisch et al., 2006) (at least five mice/genotype or treatment/time course or experiment). Total number of mouse used were as follows:‐ TRPC6(+/+) 115, TRPC6(−/−) 87, KO/*Acta2*‐TRPC6(WT) 17, KO/*Acta2*‐TRPC6(T69A) 23. Since blood flow are quickly recovered after single ligation of femoral artery in C57BL/6 background mice compared to 129Sv background (Helisch et al., 2006), ligation of femoral artery and vein followed by total excision of the superficial femoral artery and vein were performed on C57BL/6 and *Apo*E‐KO mice as described previously (Chen et al., 2017) (five mice/treatment, total number of mouse used were as follows:‐ C57BL/6 10, *Apo*E‐KO, 10). *Apo*E‐KO mice were fed with high fat diet (D12492, Research Diet, USA) for 4 weeks to induce endothelial dysfunction (Xie et al., 2006). Hindlimb hair was removed with depilatory cream and skin was cleaned by povidone‐iodine. Mice were subcutaneously injected with 0.1 mg·kg^−1^ of buprenorphine hydrochloride (Repetan; Otsuka Pharmaceutical) once after the surgery as analgesia. Hindlimb blood flow measurements were performed using Laser speckle imaging analyser (Omega wave, Japan). Under the anaesthesia described above, mice were placed supinely. Hindlimb hair was removed by depilatory cream. Laser speckle image of the ischaemic left and contralateral right legs were measured simultaneously and analysed in accordance with the manufacturer's manual. Blood flow was measured before surgery (day 0), and on days 1, 7, 14 and 21 after surgery. Blood flow recovery was expressed as a percentage of ischaemic left leg to non‐ischaemic right one. Pyr2 or CAY‐10441 were administered with mini‐osmotic pumps model 2004 (Alzet, USA) at the rate of 0.1 mg·kg^−1^·day^−1^ 3 days before or 7 days after the surgery, respectively. The dose of Pyr2 (Kitajima et al.*,* 2011; Kiyonaka et al.*,* 2009) and CAY‐10441 (Takahashi et al.*,* 2013) was determined according to the previous papers. Compounds were dissolved in DMSO and diluted with polyethylene glycol (PEG300, Nacalai, Japan). Control group (vehicle) was administered DMSO diluted with PEG300 via mini‐osmotic pumps model 2004. Cilostazol (Otsuka, Japan) was fed with normal diet at 100 mg·kg^−1^·day^−1^ calculated from average food intake 3 days before the surgery. Nω‐Nitro‐L‐arginine methyl ester (L‐NAME) was administered in drinking water at 1 g·L^−1^ 7 days after the surgery (Lloyd et al., 2001). Mice were euthanized by overdose of isoflurane at the tissue harvest.

### 2.2 Blinding and randomization

Laboratory animals were randomly assigned to experimental groups and treatments were assessed blindly. The order of treatment administration was also randomized. All animal samples were studied, and analysis was carried out in a blinded manner.

### 2.3 Measuring mRNA expression in tissues

Total RNA was isolated from frozen mouse gastrocnemius muscle samples using RNeasy Fibrous Tissue Mini Kit (Qiagen) according to the manufacturer's instructions. Quantitative real‐time PCR was performed with the ABI PRISM 7500 Real‐Time PCR System (Applied Biosystems) and OneStep RT‐PCR Kit (Qiagen) according to the manufacturer's instructions. Taqman probes (Life Technologies) used were as follows: ‐ (*Trpc* *1*: Mm00441975_m1, *Trpc* *2*: Mm00441984_m1, *Trpc* *3*: Mm00444690_m1, *Trpc* *4*: Mm00444690_m1, *Trpc* *5*: Mm00437183_m1, *Trpc* *6*: Mm01176083_m1 and *Trpc* *7*: Mm00442606_m1). Data with Taqman probe were normalized with *18S* rRNA (4352930E, Applied Biosystems).

### 2.4 Immunohistochemical analysis of mouse gastrocnemius muscles

The gastrocnemius muscles were removed from mice (five mice/genotype or treatment) and fixed overnight in 4% paraformaldehyde. After fixation, muscles were dehydrated in 10%, 15% and 20% sucrose solution, followed by embedding in optimal cutting temperature compound (Sakura finetech) and frozen in liquid nitrogen. Frozen tissues were sliced at 10‐μm slices by Leica CM1100 (Leica Biosystems). Blocking of the sections was performed with 1% bovine serum albumin for 1 hour at room temperature. Sections were stained overnight at 4°C with anti‐mouse α‐SMA (1:500 dilution, Sigma, A2547, RRID:AB_476701), anti‐CD31 (1:100 dilution, BioLegend 102501, RRID:AB_312908), anti‐NOS3 (1:100 dilution, Santa Cruz biotechnology, sc‐654, RRID:AB_631422), anti‐PTGIS (1:100 dilution Santa Cruz Biotechnology, sc‐20933, RRID:AB_2173209) and anti‐phospho‐TRPC6 (1:100 dilution) (Nishioka et al., 2011) antibodies. They were incubated with fluorescently labelled secondary antibodies (1:500 dilution, for mouse, donkey anti‐mouse IgG (H + L) highly cross‐adsorbed antibody, CF488A conjugated, Biotium 20014, RRID:AB_10561327, for rabbit goat anti‐rabbit IgG (H + L) highly cross‐adsorbed antibody, CF594 conjugated, 20113, RRID:AB_10582747 or for rat goat anti‐rat IgG (H + L) highly cross‐adsorbed antibody, CF594 conjugated, Biotium 20155, RRID:AB_10558015) for 1 h at room temperature. The specimens were observed with confocal microscope, Fluoview FV10i (Olympus, Japan) or Nikon A1 confocal imaging system with 60× oil emersion objective lens. Analysis of the number of CD31 and α‐SMA positive vessels were carried out by counting fluorescent signals in the randomly selected 20× images obtained by FV10i (three images per mouse). Clusters of CD31 immunoreactivity were counted as an individual capillary besides the morphologically identifiable vessels with a lumen. α‐SMA positive vessels were distinguished from non‐specific immunoreactivity by the presence of endothelium stained by CD31 fluorescence. Diameter of α‐SMA positive vessels were calculated from the perimeter of those in the above images. For the analysis of phospho‐TRPC6 expression in VSMCs, fluorescence signal intensity within the region of interest (ROI) being made based on α‐SMA fluorescence images was quantified, which was further normalized by the signal intensity of α‐SMA and represented as a fold increase from before surgery. Quantification of eNOS and PTGIS (prostaglandin synthase; CYP8A1) expression were carried out same as phospho‐TRPC6 except ROIs were made based on endothelial marker CD31 signal. Image J was used for all the image analysis.

### 2.5 Western blotting

Tissue samples were minced with physcotron (Microtech, Japan) in hypotonic lysis buffer (20‐mM Tris‐HCl pH 7.4, 10‐mM EDTA, 5‐mM EGTA) and proteinase inhibitor cocktail (Nacalai, Japan). Membrane fraction was precipitated with ultracentrifugation of 40,000 relative centrifugal force at 4°C for 1 h. Precipitate was dissolved in hypotonic lysis buffer supplemented with 0.1% SDS, 0.5% sodium deoxycholate and 1% NP‐40. For western blotting, the samples were fractionated by SDS‐PAGE gel and then transferred onto PVDF membrane (Millipore). The objective proteins were detected by the indicated antibody. After incubation with the secondary antibody, the bands were visualized by Western Lightning Plus ECL (PerkinElmer). Images were captured by ImageQuant LAS 4000 (GE healthcare Life Science) and quantification was performed using ImageQuant TCL software (GE healthcare Life Science). Anti‐TRPC6 (1:2000 dilution, Alomone labs, ACC‐017, RRID:AB_2040243), anti‐GAPDH (1:2000 dilution, Cell Signalling Technology, #5174, RRID:AB_10622025), antibodies were used. The Immuno‐related procedures used comply with the recommendations made by the *British Journal of Pharmacology* (Alexander et al., 2018).

### 2.6 The measurement of vascular reactivity

C57BL/6 mice were euthanized by isoflurane overdose. Immediately descending aortae were isolated and cut into approximately 1‐mm width rings. The tension measurement of the aortae was performed in Krebs solution (in mM; 130 NaCl, 4.7 KCl, 1.18 KH_2_PO_4_, 1.17 MgSO_4_, 24.9 NaHCO_3_, 5.5 glucose, 0.026 EDTA, 1.6 CaCl_2_) gassed with 95% O_2_ and 5% CO_2_ at 37°C using multi wire myograph system 620 M (DMT, Denmark). The endothelium‐dependent relaxation activity of the aortic ring was evaluated with acetylcholine (10^−9^–10^−4^ M) after pre‐maximally contracted with phenylephrine (10 μM).

### 2.7 Experimental design and statistical analysis

We predetermined the minimum sample size of 5 mice by using G*Power3.1.9.2. Since the number of pups in each litter are not always the same and to avoid any effects by the choice of pups, all the pups were analysed until the minimum sample size was satisfied in any group.

Results were presented as mean ± SEM. Sample sizes are subjected to statistical analysis at least five animal per group (n = 5), where n = number of independent values. Statistical significance was determined by Student’s unpaired *t* test for two‐group comparison and by one‐way analysis of variance (ANOVA) with Tukey's post hoc test for comparison among three or more groups. Especially, data of time courses were analysed by two‐way ANOVA followed by Tukey's post hoc test for comparison among three or more groups, or Bonferroni's post hoc test for comparison among two groups. Post hoc tests were conducted only if F in ANOVA achieved P<0.05. Significance was considered at *P* < 0.05. Statistical analysis was performed using GraphPad Prism 8.0 (GraphPad Software, LaJolla, CA). The quantification of fluorescence signal was normalized by the value before the hindlimb ischaemia surgery so that the variability of fluorescence signal in each experiment due to technical issues (i.e. antibody staining) was eliminated. The data and statistical analysis comply with the recommendations of the *British Journal of Pharmacology* on experimental design and analysis in pharmacology (Curtis et al., 2018).

### 2.8 Materials

Cilostazol (Pletal) was purchased from Otsuka pharmaceuticals (Tokyo, Japan). CAY‐10441 was purchased from Cayman Chemical (Michigan, USA). L‐NAME was purchased from WAKO (Tokyo, Japan). Pyr2 (BTP2; YM‐584893) was synthesized in Dr. Yasuo Mori's laboratory (Kyoto University) (Kiyonaka et al., 2009) and can be obtained from Tocris (Bristol, UK). Details of other materials and suppliers were provided in the specific sections.

### 2.9 Nomenclature of targets and ligands

Key protein targets and ligands in this article are hyperlinked to corresponding entries in the IUPHAR/BPS Guide to PHARMACOLOGY http://www.guidetopharmacology.org and are permanently archived in the Concise Guide to PHARMACOLOGY 2021/22 (Alexander et al., 2021).

## 3 RESULT

### 3.1 Systemic ablation of *T* *rpc* *6* facilitates blood flow recovery by increasing blood vessel maturation

**FIGURE 1:**
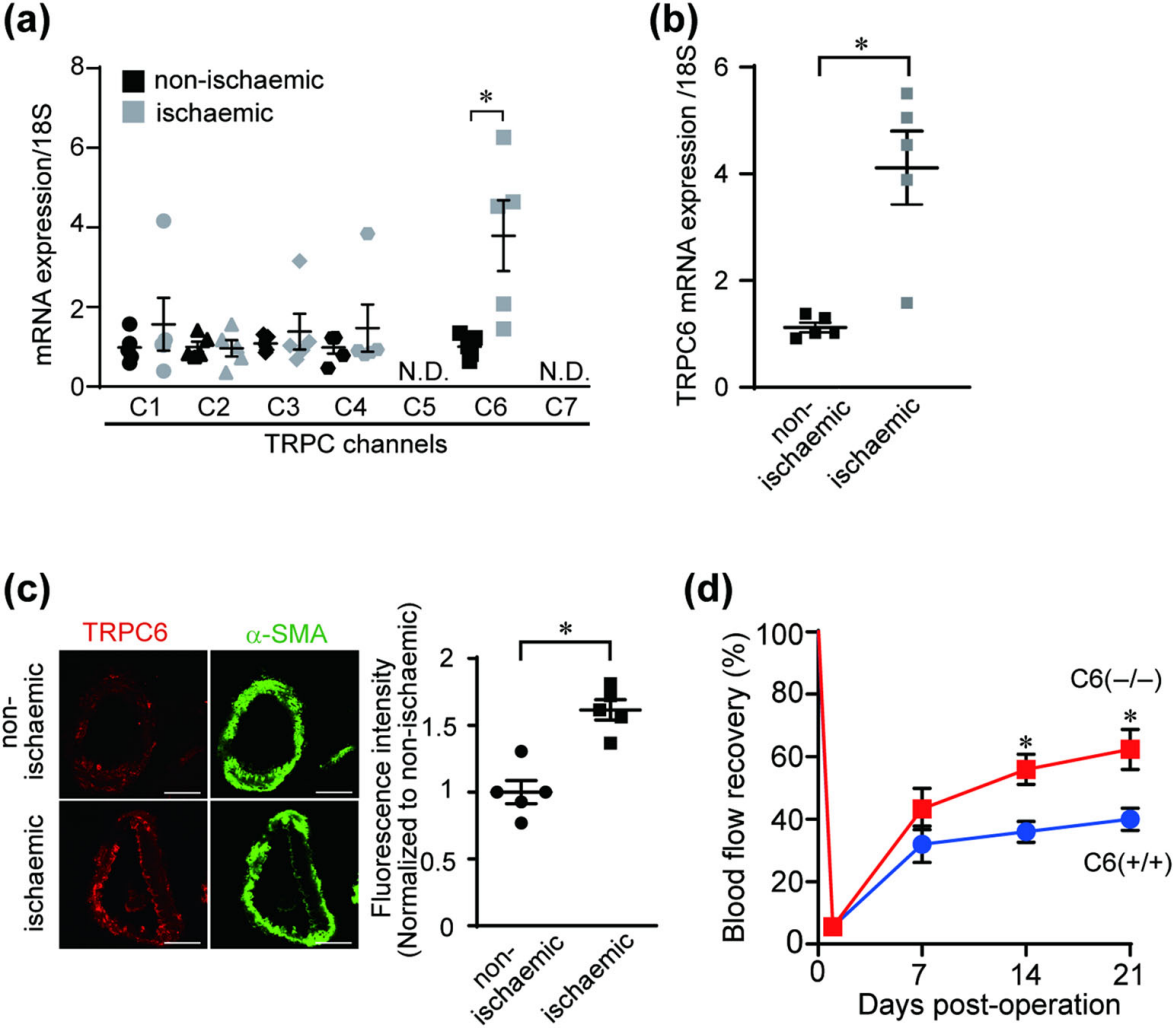
Suppression of the increased expression of transient receptor potential canonical 6 (TRPC6) facilitates the blood flow recovery after hindlimb ischaemia (HLI). (a) Quantification of *T* *rpc* *1* to *T* *rpc* *7* mRNA expressions in gastrocnemius of C57BL/6 mice 14 days after HLI (n = 5 mice per condition). N.D. denotes not detectable. (b) Quantification of *Trpc6* mRNA expression 21 days after HLI (n = 5 mice per condition). (c) Left, representative immunohistochemical images of TRPC6 and human α‐smooth muscle actin (α‐SMA) in non‐ischaemic and ischaemic gastrocnemius 14 days after HLI. Scale bars, 50 μm (left). Right, quantification of fluorescent intensity of TRPC6 over that of α‐SMA (n = 5 mice per condition). (d) Time courses of blood flow recovery after HLI in 129Sv wild‐type (C6(+/+)) and *Trpc6* deficient (C6(−/−)) mice (n = 7 mice per genotype). Blood flow before surgery (day 0) was defined as a 100% control. All data are means ± SEM. Significance was determined by unpaired *t* test (a–c) or two‐way analysis of variance (ANOVA) followed by Bonferroni's comparison test (c). *P < 0.05.

**FIGURE 2:**
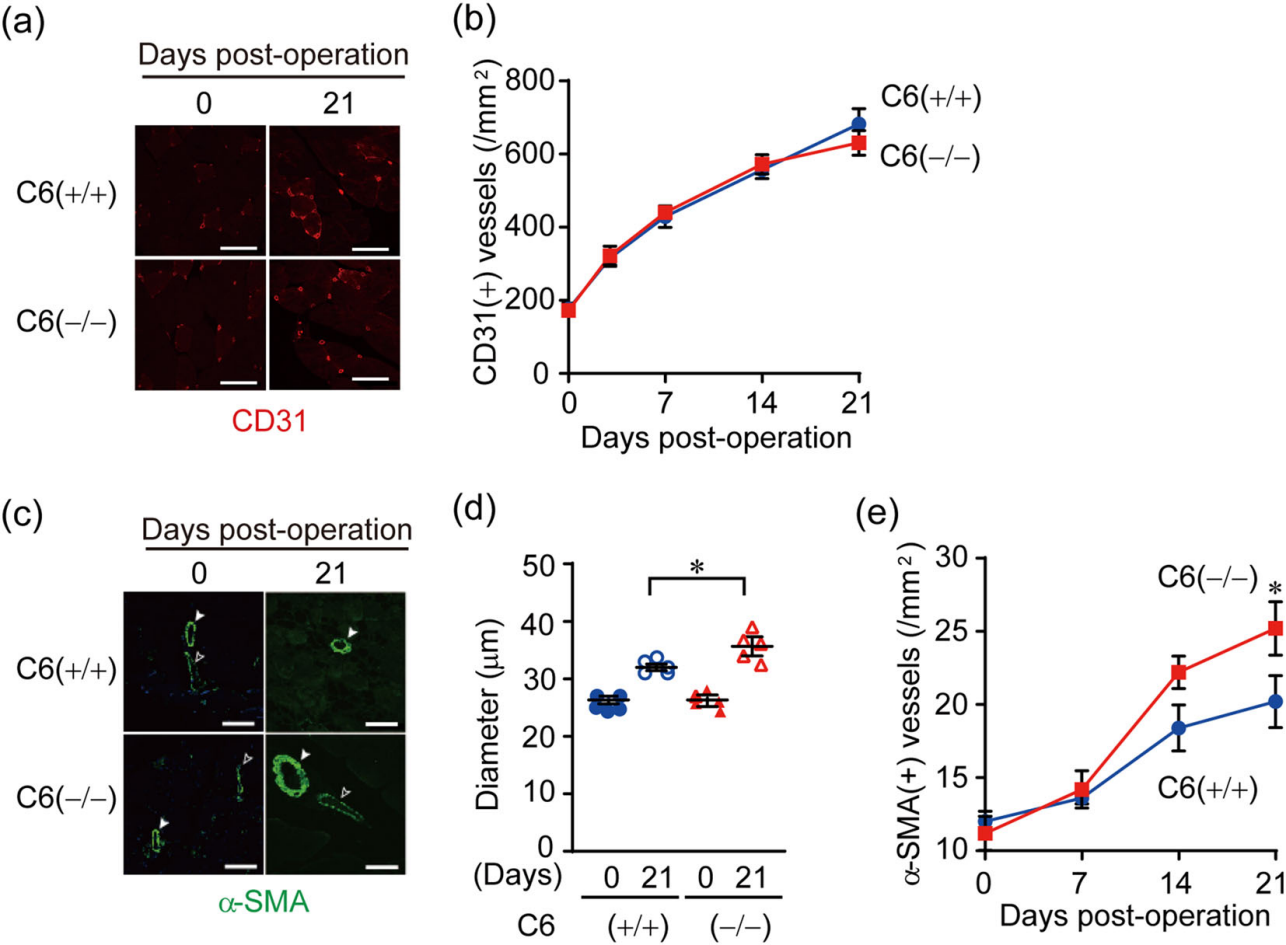
Suppression of transient receptor potential canonical 6 (TRPC6) increases vessel maturation without affecting hindlimb ischaemia (HLI)‐induced capillary formation. (a) Representative immunohistochemical images of CD31 (n = 5 mice per genotype). Scale bars, 50 μm. (b) The number of CD31‐positive blood vessels in gastrocnemius (n = 6 mice per genotype). (c) Representative immunohistochemical images of human α‐smooth muscle actin (α‐SMA). Scale bars, 50 μm. Filled and open arrow heads indicate arteries and veins, respectively (n = 5 mice per genotype). (d,e) Quantification of averaged diameter (d; n = 5 mice per genotype) and the number (e; n = 5 mice per genotype) of α‐SMA‐positive blood vessels. All data are means ± SEM. Significance was determined by two‐way analysis of variance (ANOVA) followed by Bonferroni's comparison test (b and e), one‐way ANOVA followed by Tukey's comparison test (d). *P < 0.05.

We compared the expression of *Trpc* channels in ischaemic and non‐ischaemic gastrocnemius after hindlimb ischaemia. Only *T* *rpc* *6* mRNA was increased in the ischaemic leg compared to contralateral non‐ischaemic one (Figure 1a). The upregulation of *T* *rpc* *6* mRNA was sustained for at least 21 days after hindlimb ischaemia (Figure 1b). Immunohistochemistry demonstrated that TRPC6 protein abundance was increased in the α‐SMA positive VSMCs in the ischaemic gastrocnemius (Figure 1c). These results prompted us to evaluate the blood flow recovery after hindlimb ischaemia in *T* *rpc* *6* knockout mice. *Trpc6* deficient (TRPC6(−/−)) mice showed better blood flow recovery after hindlimb ischaemia than wild‐type (TRPC6(+/+)) mice especially in the later time points of 14 and 21 days (Figure 1d). Immunohistochemical analyses revealed that the number of CD31‐positive capillaries was not different between TRPC6(+/+) and TRPC6(−/−) mice (Figure 2a,b). The number and diameter of matured vessels, which are stained by α‐SMA with CD31 were significantly increased in TRPC6(−/−) mice compared to those in TRPC6(+/+) (Figure 2c,d). Consistent with the effect of TRPC6 deficiency on the blood flow recovery, the increases of α‐SMA positive vessels were observed at later time points of 14 and 21 days post‐operation (Figure 2c–e). These data suggest that the suppression of TRPC6 facilitates the vessel maturation of newly formed capillaries induced by limb ischaemia, which gives rise to the facilitation of blood flow recovery after hindlimb ischaemia.

### 3.2 TRPC6 deletion promotes vessel maturation under the inhibition of either nitric oxide or prostacyclin production

**FIGURE 3:**
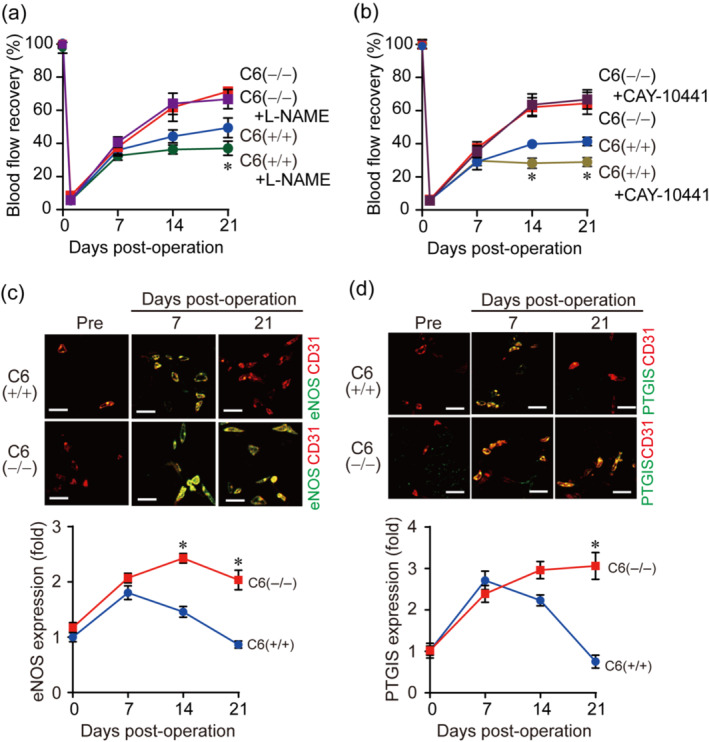
Transient receptor potential canonical 6 (TRPC6) deletion promotes blood flow recovery after hindlimb ischaemia (HLI) under the inhibition of either NO or prostacyclin production. (a,b) Time courses of blood flow recovery after HLI in TRPC6(+/+) or TRPC6(−/−) mice in the presence or absence of L‐NAME (a) or CAY‐10441 (b) (n = 5 mice per genotype and treatment). Arrow indicates the start of drug treatment. *P* values versus vehicle‐treated TRPC6(+/+) mice. (c) Representative images of eNOS and CD31 in ischaemic gastrocnemius (top). Relative temporal changes of eNOS expression in CD31‐positive vessels (bottom) (n = 5 mice per genotype). Scale bars, 20 μm. (d) Representative images of PTGIS and CD31 (top). Relative temporal changes of PTGIS (prostacyclin synthase; CYP8A1) expression in CD31‐positive vessels (bottom) (n = 5 mice per genotype). Each expression level before surgery (day 0) was defined as a control. Scale bars, 50 μm. All data are means ± SEM. Significance was determined by two‐way analysis of variance (ANOVA) followed by Tukey's comparison test (a and b), or Bonferroni's comparison test (c and d). *P < 0.05.

We next examined how suppression of TRPC6 facilitates vessel maturation in which activation of endothelial cells by shear stress plays a critical role. Nitric oxide (NO) and prostacyclin (prostaglandin I_2_: PGI_2_) are representative vasodilatory factors released from endothelial cells by shear stress and reportedly participate in vessel maturation (Otsuka et al., 2006; Park et al., 2010; Waldron & Cole, 1999). In addition, TRPC6 channel activity is also negatively regulated by its phosphorylation at Thr69 through NO‐dependent activation of protein kinase G (PKG) or PGI_2_‐dependnent activation of protein kinase A (PKA) (Nishioka et al., 2011; Shen et al., 2011; Takahashi et al., 2008). Therefore, we asked whether TRPC6 phosphorylation mediates endothelium‐dependent promotion of vessel maturation after hindlimb ischaemia. Treatment of TRPC6(+/+) mice with NOS inhibitor, L‐NAME from 7 days after hindlimb ischaemia, significantly reduced blood flow recovery, while this reduction was not observed in TRPC6(−/−) mice (Figure 3a). Inhibition of PGI_2_ receptor by the treatment with CAY‐10441 also reduced blood flow recovery after hindlimb ischaemia in TRPC6(+/+) mice, but not in TRPC6(−/−) mice (Figure 3b). These data suggest that TRPC6 inhibition can bypass the critical contribution of NO and PGI_2_ to vessel maturation during post‐ischaemic perfusion recovery. We further tested whether TRPC6 deletion enhances endothelial functions after hindlimb ischaemia. The abundances of both eNOS and PGI_2_ synthase, were transiently increased 7 days after hindlimb ischaemia but gradually declined below basal level at 21 days after hindlimb ischaemia in TRPC6(+/+) vessels (Figure 3c,d). In contrast, the increase of eNOS and PGI_2_ synthase were maintained even at 14 and 21 days after hindlimb ischaemia in TRPC6(−/−) mice (Figure 3c,d). These data suggest that inhibition of TRPC6 enhances NO and PGI_2_ production during post‐ischaemic tissue repair.

### 3.3 TRPC6 in the VSMCs negatively regulates the blood flow recovery after hindlimb ischaemia

**FIGURE 4:**
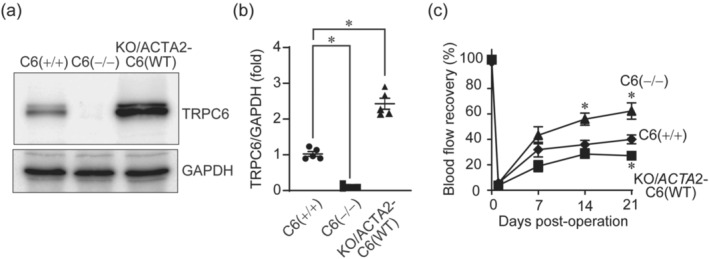
Vascular smooth muscle cell (VSMC)‐specific rescue of transient receptor potential canonical 6 (TRPC6) expression reverses the improvement of blood flow recovery after hindlimb ischaemia (HLI). (a) Representative western blots of TRPC6 and GAPDH (loading control). (b) Relative expression level of TRPC6 in KO/*Acta*2‐C6(WT) mice to those in TRPC6(+/+) and TRPC6(−/−) mice (n = 5 per genotype). The expression level of TRPC6(+/+) was defined as a control. (c) Time courses of blood flow recovery after HLI in TRPC6(+/+), TRPC6(−/−) and KO/*Acta*2‐C6(WT) mice (n = 5–6 mice per genotype). All data are means ± SEM. Significance was determined by one‐way analysis of variance (ANOVA) followed by Tukey's comparison test. *P < 0.05.

We next evaluated whether VSMC‐specific rescue of TRPC6 expression reverses the facilitating effect of TRPC6 null deficiency on blood flow recovery after hindlimb ischaemia. To specifically express TRPC6 in the VSMCs, we utilized *Acta2* promoter. The *Acta2* promoter‐driven wild‐type TRPC6 gene cassette was introduced into TRPC6(−/−) mice (KO/*Acta2*‐TRPC6(WT)). *Acta2* promoter‐driven expression of TRPC6 was analysed with lysate from aorta as a source of VSMCs. As a result, TRPC6 expression was completely rescued in KO/*Acta2*‐TRPC6(WT) mice (Figure 4a,b). The KO/*Acta2*‐TRPC6(WT) mice showed a severe retardation of blood flow recovery after hindlimb ischaemia compared to that in parental TRPC6(−/−) mice (Figure 4c), which was apparently correlated with higher expression level of TRPC6 proteins in VSMCs of KO/*Acta2*‐TRPC6(WT) mice than that of TRPC6(+/+) mice (Figure 4a,b). These data suggest that TRPC6 in the VSMCs is one of the critical determinants of blood flow recovery after hindlimb ischaemia.

### 3.4 Improvement of blood flow recovery after hindlimb ischaemia by cilostazol is correlated well with the sustained phosphorylation‐dependent suppression of TRPC6

**FIGURE 5:**
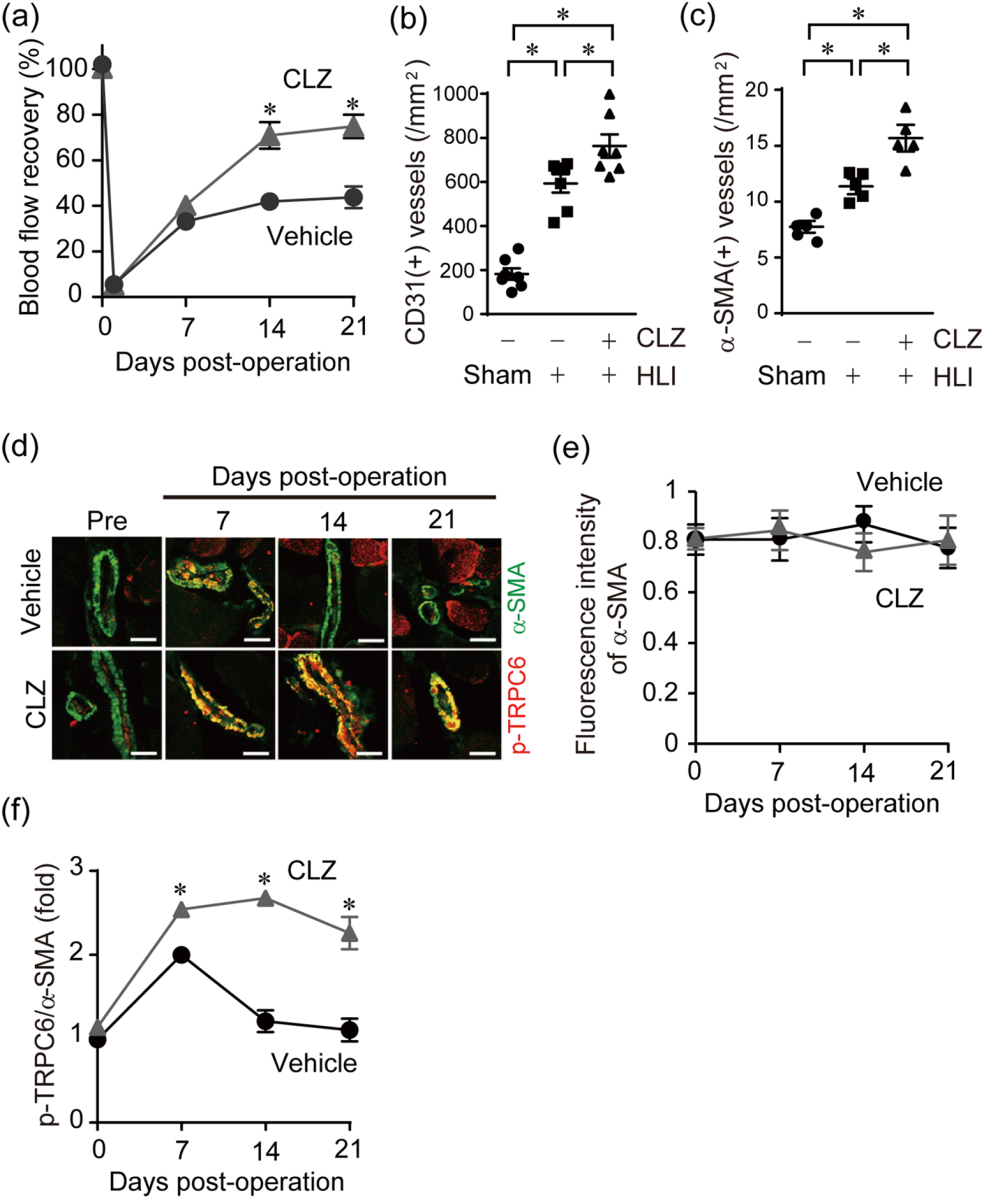
Cilostazol (CLZ) treatment improves post‐ischaemic blood flow recovery with sustained phosphorylation of transient receptor potential canonical 6 (TRPC6). (a) Time courses of blood flow recovery after hindlimb ischaemia (HLI) in TRPC6(+/+) mice treated with vehicle or CLZ (n = 6 mice per treatment). (b) The number of CD31‐positive blood vessels in gastrocnemius (n = 7 mice per treatment). (c) The number of human α‐smooth muscle actin (α‐SMA)‐positive blood vessels in gastrocnemius (n = 5 mice per treatment). (d) Representative images of Thr69‐phosphorylated TRPC6 (pTRPC6) and α‐SMA in gastrocnemius. Scale bars, 10 μm. (n = 5 mice per treatment). (e) Quantification of fluorescence intensity of α‐SMA in individual vessels (n = 5 mice per treatment). (f) Time courses of phosphorylated TRPC6 (p‐TRPC6) expression in α‐SMA positive vessels. The fluorescence intensity of p‐TRPC6 was normalized by that of α‐SMA and plotted relative to the value before surgery (n = 5 mice per treatment). All data are means ± SEM. Significance was determined by two‐way analysis of variance (ANOVA) followed by Bonferroni's comparison test (a and f) or by one‐way ANOVA followed by Tukey's comparison test (b and c). *P < 0.05.

Cilostazol is a phosphodiesterase III inhibitor that exerts an anti‐platelet and vasodilatory actions through increasing cyclic adenosine monophosphate/PKA‐dependent pathways. Cilostazol is one of the drugs known to ameliorate intermittent claudication, one of the main symptoms caused by peripheral arterial disease (Kherallah et al., 2021). In our hindlimb ischaemia model, the treatment with cilostazol significantly improved blood flow recovery after hindlimb ischaemia (Figure 5a). Cilostazol treatment increased not only the number of CD31 positive vessels (Figure 5b) but also that of α‐SMA positive ones (Figure 5c). These results suggest that cilostazol facilitated blood flow recovery after hindlimb ischaemia by increasing both angiogenesis and arteriogenesis. We previously reported that cilostazol evoked vasodilation through suppression of TRPC6 channel activity in VSMCs by PKA‐dependent phosphorylation of TRPC6 at Thr69 (Nishioka et al., 2011). Therefore, we analysed the phosphorylation levels of TRPC6 at Thr69 by the immunohistochemistry using phospho‐specific antibody of TRPC6 after hindlimb ischaemia (Figure 5d–f). α‐SMA expression remained unchanged throughout the experiment (Figure 5e). The phosphorylation of TRPC6 at Thr69 was transiently increased at 7 days and declined to the normal level at 21 days after hindlimb ischaemia (Figure 5f). The phosphorylation signals were merged well with the α‐SMA fluorescence, indicating the hindlimb ischaemia induced phosphorylation of TRPC6 in the VSMCs. The treatment of cilostazol sustained the TRPC6 phosphorylation even 21 days after hindlimb ischaemia, suggesting the correlation between blood flow recovery and phosphorylation‐dependent suppression of TRPC6 channel activity in the VSMCs.

### 3.5 Phosphorylation of TRPC6 in VSMCs is the key integrator to promote vessel maturation by cilostazol

**FIGURE 6:**
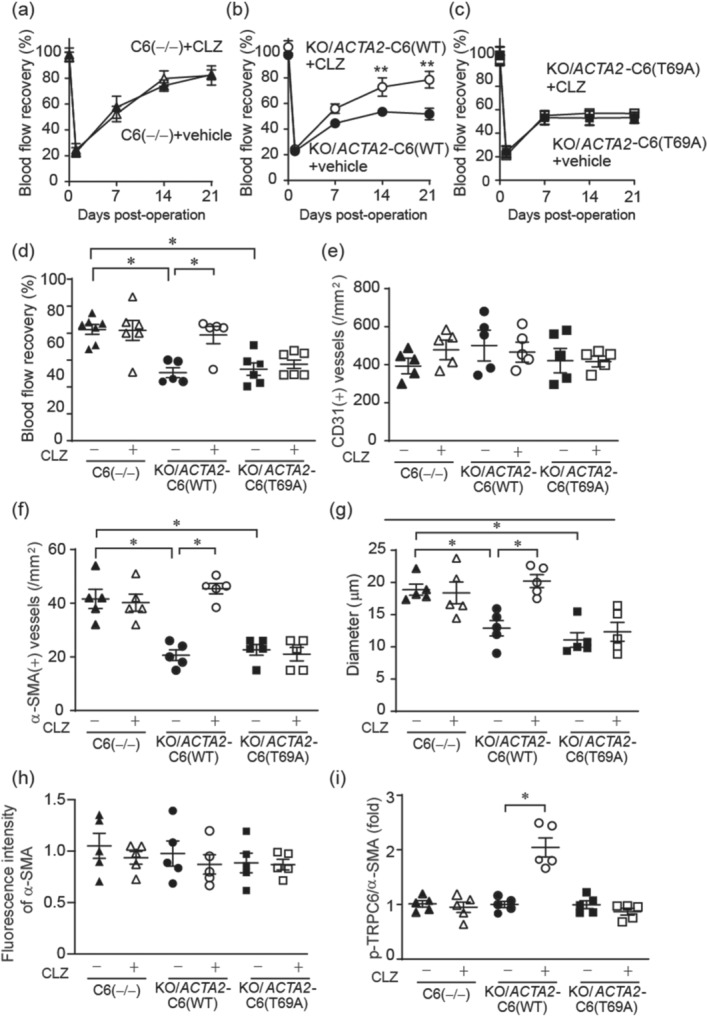
Phosphorylation of transient receptor potential canonical 6 (TRPC6) at Thr69 positively regulates arterial growth rate after hindlimb ischaemia (HLI). (a–c) Time courses of blood flow recovery after HLI in TRPC6(−/−) (a), KO/*Acta* *2*‐C6(WT) (b) and KO/*Acta* *2*‐C6(T69A) mice (c) with or without treatment of cilostazol (CLZ) (n = 6–8 mice per genotype and treatment). (d) Quantification of blood flow recovery 21 days after HLI. (n = 6–8 mice per genotype and treatment). (e) The number of CD31‐positive blood vessels in gastrocnemius from TRPC6(−/−), KO/*Acta2*‐C6(WT) and KO/*Acta2*‐C6(T69A) mice 21 days after HLI with CLZ (+) or vehicle (−). (n = 5 mice per genotype and treatment). (e,f) The number (e) and diameter (f) of human α‐smooth muscle actin (α‐SMA)‐positive blood vessels in gastrocnemius 21 days after HLI (n = 5 mice per genotype and treatment). (h) Quantification of α‐SMA expression in individual vessels 21 days after HLI (n = 5 mice per genotype and treatment). (i) Quantification of phosphorylated TRPC6 at Thr69 (pTRPC6) fluorescent intensity over α‐SMA 21 days after HLI (n = 5 mice per genotype and treatment). All data are means ± SEM. Significance was determined by two‐way analysis of variance (ANOVA) followed by Bonferroni's comparison test (a–c) or by one‐way ANOVA followed by Tukey's comparison test (d–i). *P < 0.05

Next, we tested whether phosphorylation‐dependent TRPC6 suppression underlies cilostazol‐induced promotion of blood flow recovery and vessel maturation after hindlimb ischaemia. In TRPC6(−/−) mice, treatment with cilostazol had no additive effect on peripheral blood flow recovery after hindlimb ischaemia compared to vehicle treatment (Figure 6a,d), strongly suggesting the involvement of TRPC6 in anti‐peripheral arterial disease effect by cilostazol. Cilostazol treatment significantly promoted blood flow recovery in *Acta2*‐TRPC6(WT)‐harbouring TRPC6(−/−) mice (Figure 6b,d). Furthermore, we evaluated the importance of phosphorylation of TRPC6 by cilostazol in the blood flow recovery after hindlimb ischaemia by using transgenic mice with VSMC‐specific expression of TRPC6 phosphorylation‐dead mutant, where Thr69 of TRPC6 was replaced with alanine (T69A) (Nishioka et al., 2011; Takahashi et al., 2008). The TRPC6(−/−) mice harbouring *Acta2*‐TRPC6(T69A) gene cassette (KO/*Acta2*‐TRPC6(T69A)) were subjected to hindlimb ischaemia with or without cilostazol. As a result, cilostazol treatment had no effect on the blood flow recovery after hindlimb ischaemia in KO/*Acta2*‐TRPC6(T69A) (Figure 6c,d). The recovery rates of blood flow were well correlated with the degree of the number and averaged diameter of α‐SMA‐positive matured vessels, but not with the number of those with CD31 (Figure 6e–g). The α‐SMA protein expression level in individual vessel was not significantly changed by CLZ treatment or VSMC‐specific TRPC6(T69A) overexpression (Figure 6h). Phosphorylation level of TRPC6 was significantly increased only in gastrocnemius α‐SMA‐positive vessels of *Acta2*‐TRPC6(WT)‐harbouring TRPC6(−/−) mice treated with cilostazol 7 days after hindlimb ischaemia (Figure 6i). These data demonstrated that phosphorylation of TRPC6 at Thr69 in VSMCs is a target of cilostazol during the blood flow recovery after hindlimb ischaemia.

### 3.6 Pharmacological inhibition of TRPC6 facilitates blood flow recovery after hindlimb ischaemia

**FIGURE 7:**
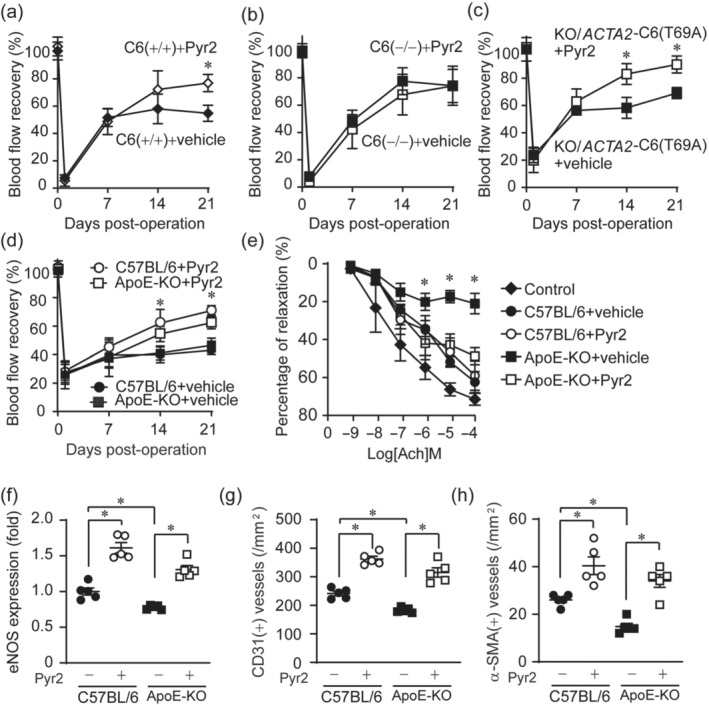
Pharmacological inhibition of transient receptor potential canonical (TRPC) channels improves blood flow recovery after hindlimb ischaemia (HLI) independently of TRPC6 phosphorylation. (a–c) Time courses of blood flow recovery in TRPC6(+/+) (a), TRPC6(−/−) mice (b) and KO/*Acta2*‐C6(T69A) mice (c) treated with Pyr2 or vehicle (n = 5 mice per genotype and treatment). (d) Time courses of blood flow recovery after HLI in control (C57BL/6) or *Apo*E‐KO mice fed with high fat diet and treated with Pyr2 or vehicle (n = 5 mice per treatment). (e) Effect of Pyr2 on the endothelium‐dependent relaxation of thoracic aorta from WT or *Apo*E‐KO mice fed with high fat diet. Aorta was pre‐contracted by phenylephrine (10 μM), and endothelium‐dependent relaxation was induced by the addition of indicated concentration of acetylcholine (ACh). (f) Quantification of eNOS fluorescence intensity (bottom, n = 5 mice per genotype and treatment). (g,h) The number of CD31‐positive capillaries (g) and human α‐smooth muscle actin (α‐SMA)‐positive vessels (h) (n = 5 mice per genotype and treatment). All data are means ± SEM. Significance was determined by two‐way analysis of variance (ANOVA) followed by Bonferroni's comparison test. *P < 0.05; NS: not significant

The above results suggested that direct inhibition of TRPC6 channel activity could be an alternative strategy for the treatment of peripheral arterial disease. We thus examined whether inhibition of TRPC channels improves blood flow recovery after hindlimb ischaemia using Pyr2. Pyr2 is a pan TRPC channel inhibitor (He et al., 2005; Kiyonaka et al., 2009). Compared to vehicle‐treated mice, Pyr2‐treated mice showed a significant increase in blood flow recovery especially at the later stage (around 21 days) after hindlimb ischaemia (Figure 7a). Same as the results of cilostazol treatment, TRPC6(−/−) mice showed no further facilitation of blood flow recovery by Pyr2 treatment (Figure 7b), suggesting that the effect of Pyr2 is solely attributable to TRPC6 inhibition. Indeed, Pyr2 treatment facilitated blood flow recovery in phosphorylation‐resistant KO/*Acta2*‐TRPC6(T69A) transgenic mice compared to vehicle‐treated KO/*Acta2*‐TRPC6(T69A) transgenic mice (Figure 7c). These results suggest that pharmacological inhibition of TRPC6 channel has therapeutic potential for peripheral arterial disease, even if endogenous TRPC6 phosphorylating activity is decreased with reduced NO availability. To test this hypothesis, we evaluated the effect of TRPC6 inhibition on blood flow recovery after hindlimb ischaemia using *Apo*E‐KO mice. Hypercholesterolemia is a critical risk factor for peripheral arterial disease (Hutter et al., 2004; Pereira et al., 2014). *Apo*E‐KO mice reportedly show the phenotype of less peripheral circulation upon hindlimb ischaemia (Couffinhal et al., 1999). *Apo*E‐KO mice were fed with high fat diet for 4 weeks to induce endothelial dysfunction (Xie et al., 2006). Pyr2 treatment significantly improved the blood flow recovery after hindlimb ischaemia even in *Apo*E‐KO mice fed with high fat diet (Figure 7d). *Apo*E‐KO aorta showed the breakdown of acetylcholine‐induced vasodilation (Figure 7e), suggesting the endothelial damage by hypercholesterolemia. Interestingly, Pyr2 treatment significantly improved endothelium‐dependent vasodilating action, suggesting that the inhibition of TRPC channel activity prevents the damage of endothelium under hypercholesterolemia. In addition, the expression levels of eNOS‐ and CD31‐positive capillaries in *Apo*E‐KO tissues were reduced (Figure 7f,g), but Pyr2 treatment significantly increased the number of eNOS‐positive capillaries and α‐SMA‐positive matured vessels in *Apo*E‐KO mice after hindlimb ischaemia (Figure 7g,h), like a phenotype of TRPC6(−/−) mice after hindlimb ischaemia (Figure 3). These results strongly suggest that TRPC6 inhibition prevents the progression of peripheral arterial disease in hypercholesterolemia model mice by facilitating capillary arterialization.

## 4 DISCUSSION

The pathophysiological role of TRPC6 in the development and remodelling of vasculature has not been well studied. In this study, we demonstrated that suppression of TRPC6 activity facilitates recovery of peripheral circulation after tissue ischaemia by using mouse hindlimb ischaemia model.

**FIGURE 8:**
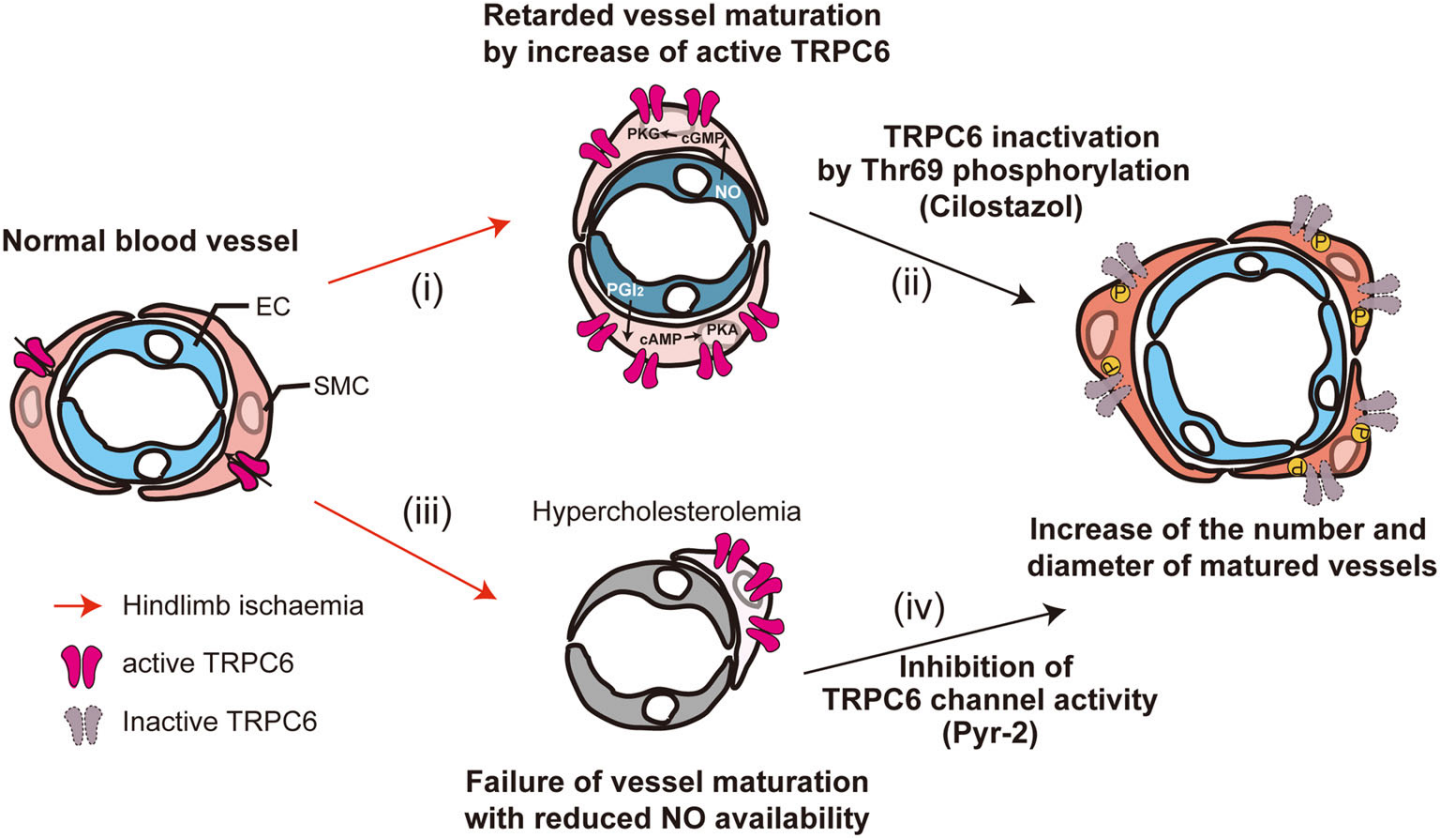
Schema of the promotion of post‐ischaemic blood flow recovery through inhibiting transient receptor potential canonical 6 (TRPC6) channel activity in vascular smooth muscle cells (VSMCs). TRPC6 channels expressed in VSMCs contribute to switching of phenotype from contractile to synthetic through cation influx. TRPC6 protein abundance is increased in VSMCs after hindlimb ischaemia (HLI), which negatively regulates vessel maturation (i). Inhibition of TRPC6 channel activity through Thr69 phosphorylation by cilostazol promotes vessel maturation and enlargement (ii). Hypercholesterolemia compromises the negative regulation of TRPC6 channel activity with reduced NO expression (iii). Pharmacological inhibition of TRPC6 channel facilitates vessel maturation even under the hypercholesterolemic condition (iv).

Based on our results, (i) hindlimb ischaemia increases TRPC6 expression in VSMCs (Figure 8). Despite the clear increase of *de novo* capillaries (such as Figure 2a,b), the blood flow in ischaemic left leg reaches a plateau around 50% compared to non‐ischaemic one 21 days after operation. The retardation of perfusion recovery is commonly observed in several mouse experiments and human patients (LeBlanc et al., 2012; Schaper, 2009). (ii) Sustained phosphorylation of TRPC6 at Thr69 by cilostazol promotes vessel maturation and enlargement through positive feedback loops between endothelial cells and VSMCs. This can increase the blood flow more than control mice (Figure 8). (iii) The reduced eNOS expression in hypercholesterolemic condition will increase the risk of peripheral arterial disease by preventing NO‐dependent phosphorylation of TRPC6 (Figure 8). However, (iv) pharmacological inhibition of TRPC6 channel activity by Pyr2 (BTP2; YM‐58483) facilitates vessel maturation even under the hypercholesterolemic condition (Figure 8).

Maturation of newly formed capillaries is one of the most important mechanisms to nurture and promote the resilience against ischaemic stress (Schaper, 2009). Vessel maturation during tissue ischaemia is mainly composed of two steps; VSMC‐proliferation dependent vessel enlargement (Schaper, 2009) and VSMC‐differentiation dependent acquisition of normal vessel function (Van Gieson et al., 2003). Many signalling molecules have been identified as critical regulators of vessel maturation using the mouse hindlimb ischaemia model (Arnold et al., 2014; Simons & Eichmann, 2015). However, loss of function of these molecules overall reduced blood flow recovery after hindlimb ischaemia. We first found that TRPC6 acts as a negative regulator of vessel maturation in mice. Suppression of TRPC6 could promote stabilization rather than formation of capillaries, which is consistent with the current idea that post‐angiogenic vessel maturation is more critical than angiogenesis for the improvement of peripheral arterial disease (LeBlanc et al., 2012). Vessel maturation after hindlimb ischaemia is a complex process that is orchestrated by several cell types, including endothelial cells, perivascular cells like pericytes, VSMCs and macrophages. In the present study, we could not find any effect of TRPC6 deficiency on angiogenesis determined by the number of CD31 positive capillaries (Figure 2a), suggesting that TRPC6 channel activity negatively regulates blood flow recovery in the later phase of hindlimb ischaemia when angiogenesis has almost finished their work. TRPC6 proteins in α‐SMA positive vessels were transiently phosphorylated but not sustained in normal mouse ischaemic leg (Figure 5d–f). These results imply that continuous inhibition of TRPC6 channel activity via phosphorylation at Thr69 is essential for efficient blood vessel maturation by perivascular cells.

The entire mechanism why TRPC6 inhibition in VSMCs promotes vessel maturation is still unclear, but one possible mechanism is suggested by our previous report that TRPC6 deficiency facilitates the differentiation of VSMCs without affecting the proliferation and migration (Numaga‐Tomita et al., 2019). The suppression of TRPC6 channel activity in VSMCs causes slight hyperpolarization upon pro‐differentiating TGF‐β stimulation, which enhances Akt activity by dissociating phosphatidylinositol phosphatase PTEN from plasma membrane. The NO dependency for the vasodilation seems different among vessel sizes. Conduit arteries are more dependent on NO than small resistance arteries in which EDHF plays a dominant role (Shimokawa & Godo, 2016). It has been demonstrated that TRPV4 channels, reportedly involved in EDH (Chen & Li, 2021; Félétou, 2016; Liu et al., 2021; Naik & Walker, 2018), play critical roles in angiogenesis and arteriogenesis after hindlimb ischaemia (Troidl et al., 2010, 2009; Yamada et al., 2021). Thus, the hyperpolarization of VSMCs is likely to be a common and important step for vessel maturation. As shown in Figure 3, two endothelium‐dependent vasodilating factors, NO and PGI_2_, contribute to the improvement of blood flow recovery after hindlimb ischaemia. Beleznai et al. (2011) report the enhanced endothelium‐dependent relaxation of mesenteric arteries from ApoE‐KO mice due to increased activity of EDH, but the blood flow recovery rate after hindlimb ischaemia was exacerbated in ApoE‐KO mice, as well as reduced eNOS expressions (Figure 7). Thus, enhancing EDH activity by inhibiting TRPC6 channel activity in VSMCs may be critical for capillary arterialization followed by blood flow recovery after hindlimb ischaemia. It is also reported that the abundance of α subunit of heterotrimeric G_q/11_ protein (Gα_q/11_)‐specific regulator of G protein signalling 5 in VSMCs promotes arteriogenesis in mice (Arnold et al., 2014). As Ca^2+^ influx through TRPC6 channels mediates Gα_q/11_‐dependent Ca^2+^ signalling in VSMCs, suppression of TRPC6 may facilitate Rho/Rho kinase‐dependent contractile differentiation and activation of VSMCs. In addition, the study of Notch ligand delta like 4 (DLL4) reveals that Notch signalling is important for post‐ischaemic perfusion recovery (Cristofaro et al., 2013). Notch signalling reportedly increases the production of PDGF and TGF‐β, which induces VSMC proliferation and differentiation, respectively (Trindade et al., 2008; Williams et al., 2006). Therefore, after ischaemia‐induced vessel expansion, how quickly the newly formed vessels are stabilized and enlarged by fully differentiated VSMCs might be critical step for the blood flow recovery after tissue ischaemia.

Inhibition of NOS activity by L‐NAME treatment significantly worsened blood flow recovery after hindlimb ischaemia, while TRPC6(−/−) mice were insensitive to L‐NAME (Figure 3a). This strongly suggests that inhibition of TRPC6 channel activity through NO/cyclic guanine monophosphate/PKG‐dependent phosphorylation at Thr69 underlies NO‐mediated blood vessel maturation after hindlimb ischaemia. Indeed, NO production is indispensable for blood flow recovery after hindlimb ischaemia demonstrated by the suppression of blood flow recovery in eNOS knockout mice or facilitation of that by administration of NO donor (Amin et al., 2012; Bir et al., 2012; Dai & Faber, 2010; Park et al., 2010). Interestingly, TRPC6 deficiency up‐regulated eNOS and PTGIS proteins in the later stage of hindlimb ischaemia, accompanying improvement of stagnant blood flow recovery (Figure 3c,d). Pharmacological TRPC6 inhibition by Pyr2 also improved endothelial function even in non‐ischaemic aorta of mice fed with high fat diet (Figure 7e). Therefore, TRPC6 inhibition not only facilitates capillary arterialization after hindlimb ischaemia, but also protects endothelium from hypercholesterolemic damage (Figure 8).

The therapeutic augmentation of collateral arterial growth in patients experiencing ischaemic diseases including peripheral arterial disease has become a challenging goal for clinical research. Cilostazol is the drug currently used for peripheral arterial disease therapy. Both facilitation of capillary formation and macrophage recruitment in ischaemic gastrocnemius vessels has been suggested as possible mechanism of action by cilostazol (Biscetti, Pecorini, Arena, et al., 2013; Biscetti, Pecorini, Straface, et al., 2013; Hori et al., 2012). In this study, we found that negative regulation of TRPC6 through phosphorylation at Thr69 also underlies promotion of vessel maturation after hindlimb ischaemia, as cilostazol had no impact on blood flow recovery after hindlimb ischaemia in rescue mice of TRPC6(−/−) expressing phosphorylated‐dead mutant TRPC6 (Figure 6). Despite high clinical utility of cilostazol, its underlying mechanism is an indirect inhibition of TRPC6 channel activity and endogenous activity of phosphorylating TRPC6 in VSMCs which could be decreased by various environmental changes, including endothelial dysfunction, hyperglycaemia and ageing. The result of our pharmacological study using a TRPC inhibitor will thus provide an alternative strategy for the treatment of peripheral arterial disease.

## CONFLICT OF INTEREST

The authors declare no conflicts of interest.

## AUTHOR CONTRIBUTIONS

M. N. supervised and conceived the project; T. N‐T., T. S. and Y. K. designed experiments and wrote the manuscript; T. S., T. N‐T., Y. K., K. N., A. N., K. S. and H.I. performed experiments and interpreted data; S. K., T. I., J. N., L. B. and Y. M. contributed reagents/analytic tools and M. N. edited the manuscript.

## DECLARATION OF TRANSPARENCY AND SCIENTIFIC RIGOUR

This Declaration acknowledges that this paper adheres to the principles for transparent reporting and scientific rigour of preclinical research as stated in the *BJP* guidelines for Design and Analysis, Immunoblotting and Immunochemistry and Animal Experimentation, and as recommended by funding agencies, publishers and other organizations engaged with supporting research.

## Data Availability

The data that support the findings of this study are available from the authors upon reasonable request.
